# Unilateral Enlarged Right Accessory Axillary Breast Tissue in a Male: A Case Report

**DOI:** 10.7759/cureus.51844

**Published:** 2024-01-08

**Authors:** Sourabh Singh, Amit Kumar, Antima Yadav, Raghvendra P Singh, Ajeet P Maurya

**Affiliations:** 1 Medicine, All India Institute of Medical Sciences, Bhopal, IND; 2 Trauma and Emergency Medicine, All India Institute of Medical Sciences, Bhopal, IND; 3 General Surgery, All India Institute of Medical Sciences, Bhopal, IND

**Keywords:** fibrous echogenic stroma, axillary swelling, fibrofatty breast tissue, adult male, accessory breast tissue

## Abstract

Accessory breast tissue, associated with polymastia and polythelia, presents challenges and concerns, particularly when patients fear malignancy. While occurring in 1-6% of cases, accessory breasts, often located bilaterally in the axillae, necessitate careful examination. We report a 35-year-old male with painful axillary swelling who underwent high-resolution ultrasonography (HR-USG) and fine-needle aspiration cytology (FNAC), revealing proper axillary breast tissue. Subsequent excision biopsy confirmed accessory axillary breast without malignancy. In conclusion, surgical removal of accessory axillary breasts is advisable, addressing cosmetic concerns and minimizing cancer risks.

## Introduction

Accessory breast tissue, known as aberrant mammary glands, is associated with polymastia and polythelia [1]. Redundant mammary glands that do not degrade or degenerate insufficiently manifest as accessory mammary glands during development. The milk line's accessory breasts are often bilateral and located in the axillae and other locations along the thoracoabdominal region. Accessory breasts are observed in approximately 1-6% of cases, with nondegraded breasts being more prevalent in females compared to males [2]. A pendulous mass or palpable thickening in the axilla may be present with axillary accessory breast. Reassurance or counseling is a must, but most of the patients with axillary breasts want surgery due to fear of malignancy. However, Accessory breast tissue cancer is a rare type of breast cancer with an incidence rate of 0.3-0.6% [3]. It typically develops in the unilateral or bilateral axilla or inguinal region, with numerous lymph nodes and multiple capillary channels.

## Case presentation

A 35-year-old male patient presented with painful swelling in the right axilla for four years; there were no signs of infection or systemic complaints. Examination revealed 3x2 cm soft, mobile, non-tender swelling in the right axilla with no overlying cutaneous pigmentation. Clinically, lipoma or enlarged lymph nodes were suspected. High-resolution ultrasonography (HR-USG), fine needle aspiration cytology (FNAC) of the right axilla, and routine blood workup were advised. The blood workup reports were within normal range (Table 1).

**Table 1:** Blood workup of the patient. AST: Aspartate aminotransferase; ALT: Alanine aminotransferase; ALP: Alkaline phosphatase.

Preoperative Values		Normal Range	Units
Complete Blood Count			
Hemoglobin	15.6	14-18	gm/dl
Hematocrit	43.2	37-47	percentage
White blood cells	9.93	4-11	Thousand/Microliter
Neutrophils	72.7	40-70	percentage
Lymphocytes	20.4	20-40	percentage
Monocytes	6.3	2-8	percentage
Basophils	0.0	0-1	percentage
Eosinophils	0.9	1-6	percentage
Platelet count	228	150-450	Thousand/Microliter
Liver Function Test			
Total bilirubin	0.6	0.3-1.2	mg/dl
Direct bilirubin	0.1	<0.2	mg/dl
AST	43	<50	U/L
ALT	46	<50	U/L
ALP	85.4	30-120	U/L
Total protein	7.57	6.6-8.3	gm/dl
Serum albumin	4.88	3.5-5.2	gm/dl
Renal Function Test			
Serum creatinine	0.83	0.6-1.2	mg/dl
Blood urea	24.51	20-40	mg/dl
Random Blood Sugar	102	70-140	mg/dl

**Figure 1:**
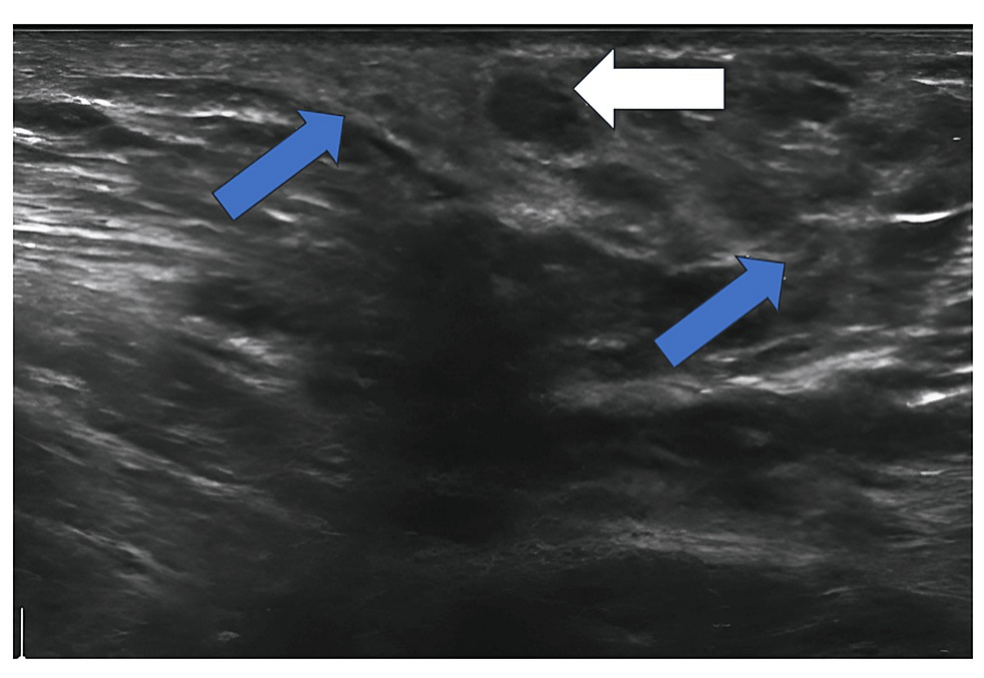
Ultrasonography of the breast tissue. The blue arrows show fibrous echogenic stroma; the white arrow shows hypoechoic glandular tissue.

HR-USG revealed a defined area of fibrous echogenic stroma with hypoechoic glandular tissue measuring approximately 3x2x1 cm in the right axilla, which was suggestive of right axillary breast tissue (Figure 1).

**Figure 2:**
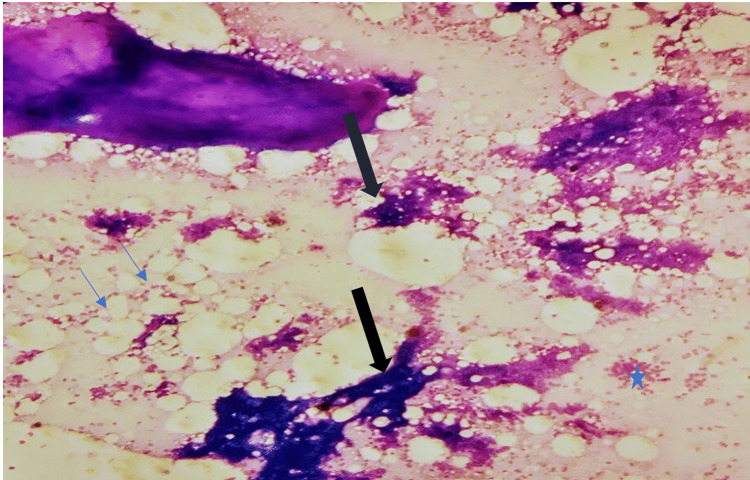
Photomicrograph of the fine needle aspiration cytology. Fine needle aspiration cytology smear at low power microscopy from the mass black arrow showing cohesive clusters of ductal cells in branching pattern, blue star showing numerous scattered bare nuclei, and blue arrows showing fat cells. (Wright stain, 50X magnification.)

A few sub-centimetric lymph nodes were also noted with maintained fatty hilum in the right axilla, the largest measuring 7.5 mm in short-axis diameter (SAD) with a cortical thickness of 2.1 mm. FNAC smears (Figure 2) revealed moderately cellular and show sheets of epithelial cells showing the bimodal population of ductal epithelial cells and myoepithelial cells along with fragments of mature adipose tissue in lipo hemorrhagic background; these features are consistent with gynecomastia, the axillary tail of breast region.

**Figure 3:**
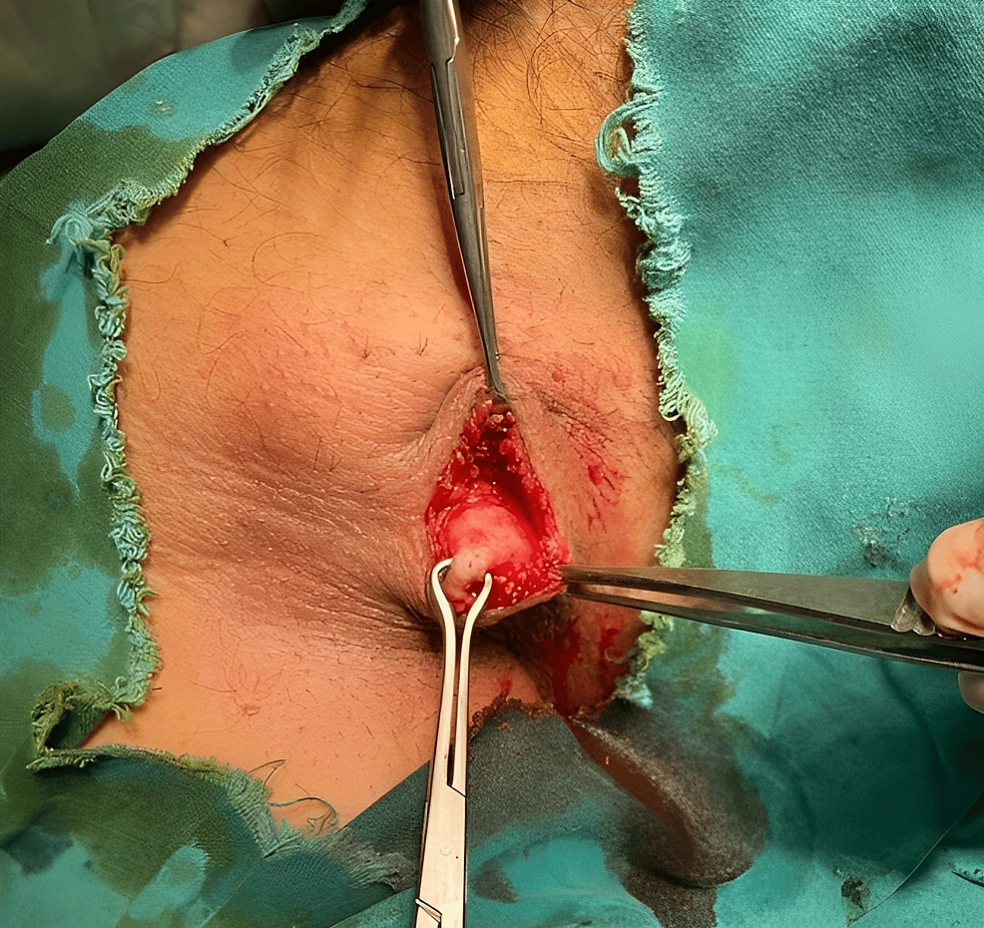
A photograph taken during surgery showing accessory axillary breast tissue being held with Babcock forceps.

After FNAC, an excision biopsy of the right axillary breast (Figure 3) was done under local anesthesia.

**Figure 4:**
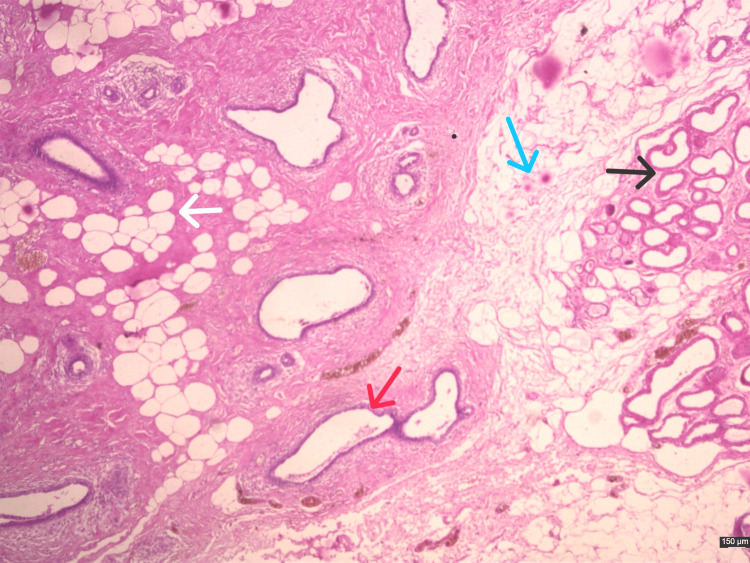
Photomicrograph depicting breast tissue. Photomicrograph of the mass showing fibrofatty breast tissue with hyalinized stroma surrounding dilated and proliferated ducts (red arrow), fat cells (white arrow), intralobular ducts (black arrow), and adjacent clusters of apocrine glands (blue arrow) seen in the dermis of the axillary skin. (H&E stain, 20X magnification.)

**Figure 5:**
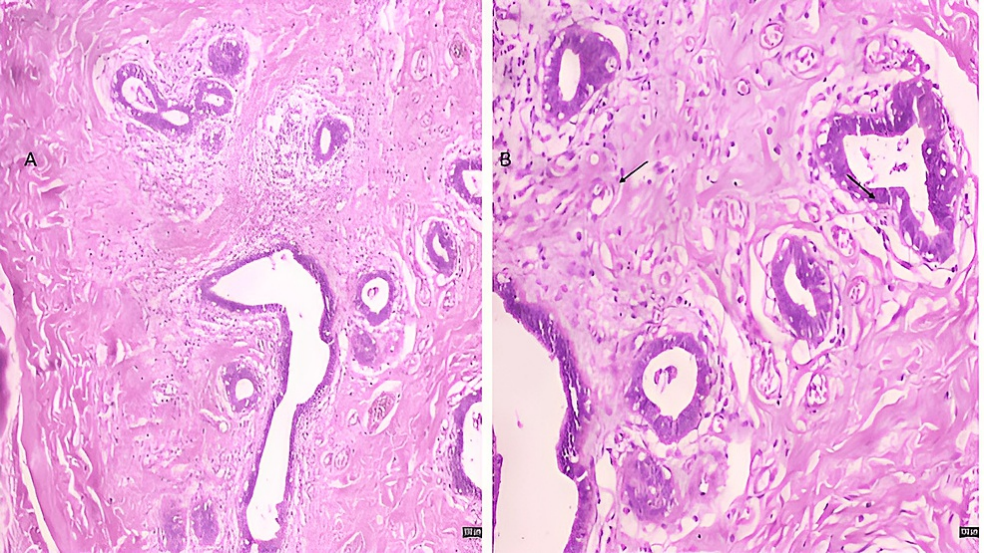
Photomicrograph A shows proliferated ducts lined by epithelial and myoepithelial cells set in a fibrous stroma, photomicrograph B shows ducts with mild epithelial hyperplasia (black arrow). 5A: H&E stain, 100X magnification; 5B: H&E stain, 200X magnification.

The procedure was uneventful, and he was managed with antacids (a tablet of 40 mg pantoprazole once daily orally before breakfast for five days), IV fluids (80 ml per hour of intravenous normal saline fluid for one day), proper antibiotics (tablet of amoxicillin and potassium clavulanate 625 mg thrice daily for five days), and adequate analgesia (tablet of diclofenac 50 mg two times daily for two days, then as per needed). He was discharged on day 3, postoperatively. On a postoperative day, ten sutures were removed. The excised specimen was sent for histopathology examination (Figures 4, 5), which revealed fibro adipose tissue with the presence of terminal duct lobular units with cystically dilated acini and focal mild epithelioid; there was no sign of atypia or malignancy, final impression was accessory axillary breast.

After three months of follow-up, the patient had no complaints and regained his normal day-to-day activities.

## Discussion

During the fifth week of pregnancy, the mammary ridge, an ectoderm, thickens and becomes the breasts. The milk line, a mammary ridge, extends from the axilla to the groin. Around 6-8 pairs of primordial breasts may develop from the axilla to the groin and inner thighs during the human embryonic phase. The mammary ridge completely vanishes throughout the subsequent weeks of gestation, except for the two pectoral regions, which develop into typical twin pectoral breasts. Anywhere else along the milk line where this mammary ridge remains could develop accessory breast tissue. When there is dysplasia, the primordial breasts that must have deteriorated persist as numerous breasts. Kajava was the first to categorize many types of accessory breast tissue in 1915. This classification, however, has been condensed to include polythelia, polymastia, and abnormal breast tissue [4]. Among women, 60% to 70% have accessory breast tissue within the milk line, and 20% have accessory breast tissue in the axilla. However, the incidence of the accessory axillary breast in males has not been reported yet; it happens to men exceptionally infrequently [5].

All the disorders that may afflict the normal pectoral breast tissue, including mastitis, abscess formation, cyclical mastalgia, milk fistula, breast cysts, fibroadenoma, fibroadenomas, fibroadenolipomas, phyllodes tumors, and rarely carcinoma, can arise in accessory breast tissue [6]. Axillary lymphadenopathy, lipoma, suppurative hyderadenitis, and sebaceous cyst are all possible diagnoses in a male with axillary swelling. The presentation of patients with accessory breasts is variable and may or may not have any symptoms. Still, some may experience pain and swelling or have concerns about the possibility of malignancy or cosmetic reasons. Although fine needle aspiration cytology provides a confirmed diagnosis, ultrasound of axillary swelling can provide a tentative diagnosis. The normal lobules, stroma, and duct may not be well structured in supplementary breast tissue. Accessory breast cancer is a rare type of breast cancer with an incidence rate of 0.3-0.6%. In a study done by Pang et al., which included 16 male patients with accessory breast tissue cancer, out of 16, 11 patients had right axillary breast tissue cancer; three patients had left axillary breast tissue cancer, one patient had right lower abdomen accessory breast tissue cancer, and another one had left lower abdomen breast tissue cancer [3]. It typically develops in the axilla or inguinal region, with numerous lymph nodes and multiple capillaries [3]. In 1957, Russia reported the first instance of male accessory breast cancer [7].

On mammograms and ultrasounds, tissue with an appearance similar to glandular tissue is shown to be present in accessory breast tissue. But this tissue shouldn't be joined to the ipsilateral breast tissue, also known as the axillary tail of Spence. MRI T1 and T2 signals are identical in accessory breast glands and regular glands.

If accessory axillary breast is asymptomatic, there is no need for treatment, and the primary justification for seeking surgical consultation is cosmesis. Due to a limited understanding of the lymphatic drainage of ectopic breast tissue, the risk of damaging nearby nerves, and developing lymphedema, it is advisable to remove symptomatic tissue carefully [8]. Nowadays, plastic surgeons also perform axillary breast liposuction [9].

Various potential diagnoses may arise when a man exhibits symptoms of axillary swelling, such as lipoma, suppurative hidradenitis, sebaceous cyst, or axillary lymphadenopathy. Investigating individuals presenting with these symptoms is imperative, as accessory axillary breasts carry a heightened risk of cancer compared to typical breasts. Thus, comprehensive assessments are essential to accurately identify and address underlying conditions, ensuring timely and effective management.

## Conclusions

In conclusion, individuals with axillary accessory breast tissue should consider the surgical removal of these breasts due to the relatively safe nature of the procedure and the alleviation of concerns regarding potential malignancy. This procedure significantly contributes to the patient's physical and psychological well-being.
